# Economic efficiency of primary care for CVD prevention and treatment in Eastern European countries

**DOI:** 10.1186/1472-6963-13-75

**Published:** 2013-02-23

**Authors:** Titus Slavici, Claudiu Avram, Gabriela Victoria Mnerie, Adriana Badescu, Doina Darvasi, Florin Molnar-Matei, Mihai Aristotel Ungureanu

**Affiliations:** 1 Politehnica University of Timisoara, Timisoara, Romania; 2 Department of Economics, Ioan Slavici University, Timisoara, Romania; 3 Physical Education and Sport Faculty, West University of Timisoara, Romania; 4 Faculty of Internal and International Commercial and Financial-Banking Relations, Romanian American University, Bucharest, Romania

**Keywords:** Cardiovascular diseases, Prevention, EUROASPIRE III Romania Follow Up, Economic burden, Cost improvement

## Abstract

**Background:**

Cardiovascular disease (CVD) is the main cause of morbidity and mortality worldwide, but it also is highly preventable. The prevention rate mainly depends on the patients’ readiness to follow recommendations and the state’s capacity to support patients. Our study aims to show that proper primary care can decrease the CVD-related morbidity rate and increase the economic efficiency of the healthcare system.

Since their admission to the European Union (EU), the Eastern European countries have been in a quest to achieve the Western European standards of living. As a representative Eastern European country, Romania implemented the same strategies as the rest of Eastern Europe, reflected in the health status and lifestyle of its inhabitants. Thus, a valid health policy implemented in Romania should be valid for the rest of the Eastern European countries.

**Methods:**

Based on the data collected during the EUROASPIRE III Romania Follow Up study, the potential costs of healthcare were estimated for various cases over a 10-year time period. The total costs were split into patient-supported costs and state-supported costs. The state-supported costs were used to deduce the rate of patients with severe CVD that can be treated yearly. A statistical model for the evolution of this rate was computed based on the readiness of the patients to comply with proper primary care treatment.

**Results:**

We demonstrate that for patients ignoring the risks, a severe CVD has disadvantageous economic consequences, leading to increased healthcare expenses and even poverty. In contrast, performing appropriate prevention activities result in a decrease of the expenses allocated to a (eventual) CVD. In the long-term, the number of patients with severe CVD that can be treated increases as the number of patients receiving proper primary care increases.

**Conclusions:**

Proper primary care can not only decrease the risk of major CVD but also decrease the healthcare costs and increase the number of patients that can be treated. Most importantly, the health standards of the EU can be achieved more rapidly when primary care is delivered appropriately.

**JEL:**

I18, H51

## Background

### CVD: the present situation

Cardiovascular diseases (CVDs) are non-communicable diseases that affect the cardiovascular system (i.e., the heart and blood vessels). These diseases include heart attack and stroke.^a^ CVDs are ranked as the leading cause of mortality and morbidity in the world. The CVD-related mortality rate ranges from 4% in high-income countries to 42% in low-income countries [1]. The mortality rates from stroke are higher in Central and Eastern Europe than in Northern, Southern and Western Europe [2]. Overall, CVD is estimated to cost the EU economy 192 billion EUR a year, 57% of which is due to healthcare costs, 21% to productivity losses and 22% to informal care [3]. However, with proper primary care, CVDs are highly preventable [1,3].

The identified risk factors for CVDs include the following: heredity, socio-economic changes, cultural changes and behaviour. To a certain extent, one can decrease or increase the risk of CVD by modifying these factors. While heredity cannot be changed and the socio-economic situation is difficult for an individual to change, individual behaviour can easily be modified and doing so can yield good results for CVD prevention.

There are many consequences of CVD. For the individual, these consequences can range from death to dramatic changes in lifestyle (e.g., disability and decreased productivity), increased costs of long-term medical treatment and rehabilitation and even impoverishment. For the community, an individual recovering from CVD necessitates managing a less productive worker and supporting extended healthcare costs because part of the treatment costs are supported by the state.

There are two methods of treating CVDs: treatment of the risk factors that are present before a severe CVD occurs and treatment of the consequences of a severe CVD. Treating (or managing) the risk factors implies prevention of the disease (i.e., primary care). Countries that have implemented prevention programs have reduced the rate of CVDs in the last two decades [1,3]. Treating the effects implies treating the disease and managing its consequences as they occur. This practice prevails in countries that have not implemented a prevention program. Economic studies have been performed both to evaluate the effectiveness of combat strategies and interventions in low- and middle-income countries [4] and to provide a better method of resource allocation [5].

The cultural differences between Western^b^ and Eastern^c^ Europe were deepened by the socio-economic and political development of both regions during the last century [6]. Western European countries have been more successful in implementing health policies than Eastern European countries have and the national health indicators therefore reflect a healthier population with a healthier lifestyle and healthier dietary habits. Additionally, there is a significant difference in lifestyle habits and dietary habits between countries in the west and east. The Eastern European countries, which have a poorer lifestyle and dietary habits, do not score well on many national health indicators [3]. Once admitted to the EU, an Eastern European country is committed to implementing the Union’s policies, thus it must implement the Union’s health policies and achieve the health standards of the Union.

The socio-economic situation in Romania is similar to that of other Eastern European countries and the average Romanian faces the same challenges and the same health problems as the average Eastern European. This situation has a direct influence on the habits of the population and hence the national health indicators in Romania are representative of Eastern European countries as a whole. Thus, the effects of a health policy on the average Romanian are similar to the effects on the average Eastern European individual.

Even if Romania is viewed as a developed country [7] and the health status indicators present a positive trend, these indicators remain below the EU and regional averages. In Romania in 2010, 3466547 individuals from a total population of 21431298 were affected by various forms of CVD (16175.16 per 100000 population). Additionally, 90608 people died from a CVD (422.78 per 100000 population). Out of 259723 deaths in Romania in 2010, 34.89% were caused by a CVD [8,9]. This figure can be compared with constantly time-decreasing CVD mortality rates of less than 200 per 100000 population in countries such as France, Spain, the Netherlands, Italy, Denmark and Norway [1]. Another indicator of the CVD burden is the percent of the total healthcare costs allocated to CVDs [3]: in France and Spain, only 7% of the individual healthcare costs are generated by CVDs; in Cyprus and Denmark, this percentage is the lowest (5%); however, in Romania and Estonia, 15% of the individual healthcare costs are generated by CVDs. The only EU country with a worse status is Poland (17%).

The economic impact of stroke, including its psychological and social aspects, was studied in the Netherlands [10]. Little information about the effectiveness of physical activity enhancement strategies is provided for developing countries, while this information is highly documented in developed countries [11]. In 2006, the economic burden of CVD in the enlarged EU was investigated [12]. Although that study has important limitations, it found that productivity losses and informal care represented 21% and 17% of CVD-related costs, respectively.

Since signing the European Heart Health Charter in 2007, Romania has been committed to fighting CVD and its effects [13]. The goals of this campaign are to reduce the morbidity and mortality rates to levels comparable to the EU averages: under 4000 per 100000 population for morbidity and under 400 per 100000 population for mortality. However, because the morbidity and mortality rates do not decrease dramatically overnight, the achievement of these goals takes a period of time that depends on the number of people who pay attention to their heart health. An additional issue is the problem of coverage (i.e., the number of patients with severe CVD that can be treated given the healthcare budget). With a fixed healthcare budget, the rate of coverage should increase as the CVD-related morbidity rate decreases.

The economics of primary care was investigated in [14] and the conclusion was that little is known about the economic impact of health promotion interventions compared to clinical prevention activities. The authors also highlighted the importance of governmental engagement in economic evaluations of prevention activities.

Our study aims to document the economic importance of CVD prevention compared with treatment of the disease. To this end, we considered the database of the EUROASPIRE III Romania Follow Up and we computed the costs in the following manner. The minimum costs in the case of a fatal heart disease were estimated for different scenarios that corresponded to various levels of both the patient’s interest in his/her health state and the severity of the CVD consequences. At the beginning of the study, the Heart SCORE [15] was computed, yielding the probability of fatal heart disease for the next 10 years. The lowest standardised cost of disease was computed as the minimum cost of heart disease multiplied by the SCORE probability. Then, the prevention costs over a period of 1.5 years were estimated. The SCORE probability was again computed after a period of 1.5 years. The lowest standardised costs of fatal heart disease were estimated for the remaining 8.5 years. These values were compared with the number of recommendations followed by the patients. Our approach emphasises that with a proper primary care program, there is no need to identify (or allocate) additional resources for the enhancement of the healthcare budget.

## Methods

### Data collection and usage

The medical data concerning the effect of the prevention policies on Romanian CVD-patients were recorded during the EuroAspire III Romania Follow Up [16-18]. All the patients participating at this study were voluntary. They understood and agreed that their medical records obtained during the follow up should be anonymously used for medical and statistical purposes. Furthermore, the research team involved in the medical study cooperated with a team of economists from the Department of Economics of the Ioan Slavici University of Timisoara, in order to obtain an economic point of view on the various policies of CVD management.

The statistical data for Romania were collected from public databases: [2,8,9,19,20].

Based on the above-presented data, the best possible CVD-related scenarios were created (i.e., the least damaging consequences were considered, thus producing minimal costs of care). Of course, more serious consequences of severe CVD (i.e., permanent disability or the need for a highly specialised intervention) can only increase costs and thus reinforce our conclusions. Thus, the economic impact of CVD for different groups of patients was described and the economic effects of various healthcare policies were determined, both for the patient and for the state.

### Causes and effects of CVDs considered in the present study

The commonly cited risk factors for CVD include stress, high blood pressure, elevated blood glucose and blood lipid levels and obesity. However, these are mostly secondary causes of disease. The primary causes of CVDs are heredity, socio-economic and cultural changes and individual behaviour.

Heredity is a non-modifiable class of factors that includes the following: age (older people are more predisposed to CVDs), sex (men are more predisposed than women) and family history. Another class of factors is represented by socio-economic and cultural changes. These factors cannot be modified in the short term and depend mainly on the community in which one lives. These factors include globalisation, urbanisation, population ageing, impoverishment, pollution and others. The main consequence of these factors is stress. Individual behaviour comprises diet, physical activity and individual habits such as smoking and alcohol drinking. These factors are the easiest and least costly to modify. They can be modified in a relatively short amount of time, but that depends mainly on an individual’s options. Unhealthy diet, lack of physical activity, smoking and alcohol abuse cause high blood pressure, increased blood glucose levels, increased blood lipid levels, weight gain and obesity. The direct influence of physical activity on health is underlined in [21,22]. Without modification, one or more of these secondary risk factors can lead to the development of CVD. Hence, the present work analyses mainly the effects of changing these secondary risk factors on the patient’s health (prevention).

The effects of CVDs vary and range from temporary disability to death. With the exception of death, all the consequences to the patient involve a decrease in productivity. A patient recovering after a severe CVD requires help for routine activities, which vary from housekeeping to personal assistance. In addition, because of the productivity decrease, income decreases for patients who are not retired. The long-term care expenditures for European countries were evaluated [23], but the European countries studied were Germany, Italy, Spain and the United Kingdom. No Eastern European country (including Romania) was considered in this study. An economic evaluation of cardiac rehabilitation was performed in [24]; however, due again to the lack of available data, no Eastern European country was considered. This lack of information concerning Romania as an Eastern European country will be (partly) addressed by our current study.

### The EUROASPIRE III Romania follow up

The EuroAspire III Romania Follow Up study started in November 2007 as a continuation of EuroAspire III Primary Care [16-18] in Romania and its primary goal was the implementation of the European prevention recommendations for individuals with high cardiovascular risk in a geographic region where the incidence of CVD is very high largely because of unhealthy lifestyle. The aims of this study included the following: 

•to expand lifestyle modification interventions offered by primary care physicians using the ESC Prevention Kit to reduce the risk of CVD in high-risk asymptomatic patients;

•to control cardiovascular risk factors through the proper use of medications to achieve the targets recommended by the European Guidelines for the Prevention of CVD;

•to recommend selective cardioprotective medications by primary care physicians in collaboration with cardiologists, endocrinologists and nephrologists; and

•to create a model of change that is applicable to other centres in Romania and Europe.

The 18-month follow-up study was conducted with 325 voluntary patients who took part in EuroAspire III Primary Care Romania. The patients were recruited consecutively from primary care offices from 6 months to 3 years after their diagnosis and treatment initiation. The inclusion criteria included the following: high cardiovascular risk, under 80 years of age, no history of coronary or other atherosclerotic disease, and the use of the following types of medications: 

•antihypertensive drug therapy;

•lipid-lowering drug therapy; and/or

•diabetes therapies (diet and/or oral hypoglycemic and/or insulin).

The inclusion criteria correspond to the following risk factors: high blood pressure (HBP), high blood sugar (HBS), or high blood lipids (HBL) [25,26]. The classification of patients was performed according to the European standards [27,28], which include the definition of high CV risk, high blood pressure, high glucose and blood lipids values. Independently of the study protocol, 23 patients (6.6% from the patients initially asked) refused to participate to the follow up. Major cause of refusal to participate was the lack of availability to participate to the regular visits on 6 months, mainly because of traveling or changing the city or country of residence during the follow up period. No significant differences in term of age, gender, level of CVD risk was noticed between those who volunteered to participate and those who refused. More detailed data are given in Table 1. The ratio of each group of patients corresponds to the national ratio of CVD patients.

**Table 1:** EUROASPIRE III follow up Romania characteristics

**Characteristic**	**Male**	**Female**	**Total**
Test Population	123	202	325
Inclusion criteria: HBP	35	67	102
Inclusion criteria: HBL	8	28	36
Inclusion criteria: HBS	2	0	2
Inclusion criteria: HBP and HBL	57	83	140
Inclusion criteria: HBP and HBS	6	9	15
Inclusion criteria: HBP and HBL and HBS	15	15	30
Age (interval)	30 - 75	27 - 78	27 - 78
Age (average)	57.983 ± 8.748	57.802 ± 8.783	57.870 ± 8.757
Work status: employee (full time or part time)	69	76	145
Work status: family worker, unemployed or undeclared	3	7	10
Work status: retired	51	119	170
Initial SCORE (interval)	1 - 26	1 - 19	1 - 26
Initial SCORE (average)	7.360 ± 6.394	3.247 ± 2.769	4.796 ± 4.906
Final SCORE (interval)	1 - 18	1 - 13	1 - 18
Final SCORE (average)	5.393 ± 3.987	2.465 ± 1.993	3.567 ± 3.232
P-value	0.00205	0.000609	0.000081
Null hypothesis (the reduction of SCORE is not statistically significant)	REJECTED	REJECTED	REJECTED

Characteristics of patients involved in the Euroaspire III Follow Up Romania. Note that the reduction of SCORE is statistically significant at a confidence level of 99%.

The primary care physicians were trained by an interdisciplinary team (cardiologist, endocrinologist, nephrologist) to reinforce lifestyle changes (ESC Prevention Kit) and to optimise medications according to the current clinical and biochemical parameters (ESC Prevention Guidelines) [28].

For all the patients, the HeartScore was computed at the beginning and at the end of the prevention program. The SCORE system is a better instrument for evaluating CVD risk than the Framingham test. It takes into account various risk factors (sex, age, smoking status, blood pressure, cholesterol, diabetes and family history) and computes the probability (in percentages) of experiencing a fatal CVD in the next 10 years [15]. For our study, the HeartScore varied from 1 to 26 at the beginning of the programme and from 1 to 18 at the end of the programme.

At the beginning of the programme, the risk factors and the health status of the patients were evaluated by routine analyses. The patients received appropriate lifestyle recommendations [28,29] and medications from their physicians to reduce their cardiovascular risk. The health status of the patients was re-evaluated every 6 months and the lifestyle recommendations and medications were modified accordingly. After 18 months, a final evaluation was performed and the SCORE factor was computed again.

From 325 total patients receiving cardioprotective medications, 171 (52.61%) received recommendations to follow a rehabilitation program (basically regarding their lifestyle and physical activity). However, only 44 of these 171 patients (25.73%) completed at least half of this program. Additionally, only 296 of the 325 patients (91.07%) regularly complied with the medication regimen (i.e., they did not forget to take the medications (but at most rarely) and did not change the medication dosage (but at most rarely)). This class of patients received a proper primary care treatment regimen and thus our study is based on this class of patients.

### Costs

In the following model, the estimated costs are expressed in Romanian currency (RON) at the 2010 level. The corresponding costs expressed in EUR could be obtained by taking into account the average exchange rate at the 2010 level: 1 EUR = 4.2099 RON [20]. To extend the costs to a 10-year time period, the 2010 costs are updated by taking into account a value of 5% for the Consumer Price Index (CPI).

#### Prevention costs

To prevent CVDs, one must modify their causes. Prevention (i.e., primary care) includes the following: 

•regular visits to the general practitioner (family physician);

•regular routine blood analysis;

•inexpensive medication; and

•inexpensive lifestyle and diet modifications.

A regular visit to the family physician is defined as a 30-minute visit every 6 months during which routine analyses are performed and recommendations (lifestyle and medication) are prescribed. Such a visit is estimated to cost 50 RON, of which 10 RON is normally supported by the patient.

Regular routine blood analysis measures the levels of blood glucose and blood lipids. These analyses are performed once every 6 months and cost 40 RON, half of which is supported by the patient.

The monthly medication is directed at three of the secondary causes: high blood pressure, high blood glucose and high blood lipids. Antihypertensive medication has a monthly cost of approximately 150 RON, of which 50% is supported by the patient and 50% supported by the state. Antihyperlipidaemic medication costs approximately 150 RON monthly - 50% of which is supported by the patient. Diabetes medication is estimated at 50 RON monthly and this cost is entirely supported by the state.

Lifestyle modifications comprise the following: improving the diet, increasing the physical activity and, for smokers, reducing the number of cigarettes smoked. The diet is significantly improved by reducing the number of calories consumed; reducing the fats, sugar and salt in the daily menu; consuming fruits and vegetables daily; and consuming fish weekly. These changes are rather inexpensive^d^.

#### Treatment costs for acute forms of CVD

The risk of a major CVD, such as heart attack or stroke, increases when prevention measures are not effective. In this case, additional activities include the following: 

•emergency care, which includes hospitalisation, specialised analysis, medical intervention and emergency medication;

•regular visits to specialists such as cardiologists, diabetes specialists and endocrinologists;

•regular investigations;

•post-traumatic long-term medication; and

•post-traumatic long-term rehabilitation.

In cases of severe CVD, emergency care includes the following: hospitalisation costs (566 RON per day at a cardiology clinic at an average of 7.4 days of hospitalisation entirely supported by the state in the case of insured patients); emergency medication costs (estimated at 850 RON, 10% of which is supported by the patient); and intervention costs (estimated at 2125 RON, 50% of which is supported by the patient).

Because CVDs often cause various levels of disability, long-term post-traumatic rehabilitation and care is often required and the associated cost is estimated at 1000 RON monthly, which is entirely met by the patient.

The cost of post-traumatic long-term medication is estimated to be approximately twice the cost of preventive medication.

The visits to specialists are estimated at 100 RON per visit. After a stroke or a heart attack, one must consult the specialist after 1 month, after 3 months and then every 6 months.

The special investigations are estimated at 300 RON and are to occur with the same frequency as the visits to the specialists.

The long-term medication is approximately 1.5 times as expensive as the preventive medication and is supported half by the patient and half by the state.

The post-traumatic long-term rehabilitation may include the following: help from family members or specialised care providers, yearly treatments in rehabilitation clinics and kinesiology.

In the case of permanent disability, the patient may receive a monthly disability pension, which 2010 Romanian laws established at 512.96 RON. In addition, permanent disability enables the patient to benefit from the services of an attendant, which is paid for by the state at a monthly wage of 586.24 RON.

The healthcare costs were computed according to the scenarios presented below. Standardised costs were also computed (i.e., the cost of healthcare multiplied by the SCORE value).

### Scenarios

**Figure 1:**
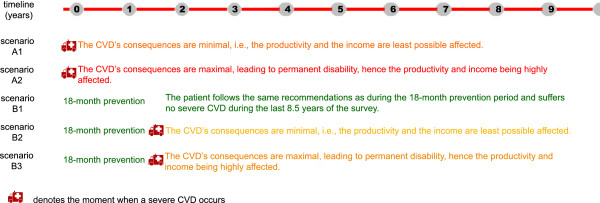
**Scenarios outline.** An intuitive outline of the scenarios considered in the present article.

There are two attitudes of patients regarding heart health: concern and disinterest. The following scenarios are constructed based on these two attitudes. An outline of these scenarios is presented in Figure 1.

#### Cost-related scenarios

The following scenarios were developed to estimate the costs of CVDs. For these scenarios, the latest data available for the morbidity and mortality rate were considered and the rate of risk reduction was computed from the EUROASPIRE III Follow Up results.

The first case (case A) represents a scenario when no primary care was ever provided to the patient. The costs are estimated for the 10-year period after the occurrence of a severe CVD. This scenario can occur when the patient either does not know his risk factors or does not follow the recommendations (and medication) received from the general practitioner. The better scenario (case A1) is the case in which the patient is subjected only to minor effects of CVD and thus spends two weeks per year in a rehabilitation clinic and benefits from two hours of daily assistance from family members. The worst- case scenario (case A2) is the case in which the patient suffers a permanent disability and needs permanent care from another person (Figure 1).

The second case (case B) assumes that an 18-month course of primary care was provided to the patient. The patient is assumed to have followed the recommendations and taken the medication received during these 18 months. This case can be further split into two subcases that depend on the scenario chosen for the next 8.5 years. The better scenario (case B1) is that no severe CVD occurs during the remaining 8.5 years and thus the costs supported by the patient (and the state) are proportional to the costs supported during the first 18 months. The average scenario (case B2) assumes that the patient experiences a severe CVD with minor consequences immediately after the 18 months of primary care and the costs are proportional to the costs generated in the case A1. The worst-case scenario (case B3) assumes that the patient experiences a severe CVD leading to a permanent disability immediately after the 18 months of primary care and the costs are proportional to the ones computed in case A2 (Figure 1).

#### Coverage-related scenarios

The two attitudes of the patient also dictate the coverage scenarios.

The first scenario (i.e., the “bad coverage scenario”) assumes that the proportion of patients concerned about their heart health is constant over time. This ratio can vary from 0% (worst possible: no one is interested in their heart health in the present or in the future 15 years) to 100% (best case possible).

The second scenario (i.e., the “good coverage scenario”) assumes that if a given proportion of patients is initially interested in their heart health, the number of patients in this cohort increases by a constant rate over time (i.e., if 30% of the patients are currently interested in primary care, then next year, an additional 30% of the remaining patients will become interested in primary care). We do not discuss the method by which the patients become interested in heart health here; we only accept that this situation is possible.

## Results and discussion

### Prevention costs versus treatment costs for severe cases

#### General costs

Based on the costs presented above, the treatment costs in severe CVD cases (cases A1 and A2) were computed for each group of patients. The detailed costs are presented in Table 2. On average, the costs are as follows: 

– emergency costs of 4188 RON, occurring once in the 10-year period;

– visits to the specialists: 22 visits (occurring at 1 month, 3 months and then every 6 months after the emergency), generating costs of 2200 RON over 10 years;

– investigations performed concurrently with the visits to the specialists, generating costs of 8360 RON over 10 years;

– medication, which was estimated to cost 300 RON monthly during the 10-year period;

– other costs: the best case possible suggests that after suffering a severe CVD, the patient needs at least 2 hours of care daily, costing approximately 248.7 RON monthly [19], which is either paid by the patient or not generated as income by one or more of his relatives; and

– each year, the patient is supposed to spend 14 days hospitalised in a specialised rehabilitation clinic, generating a yearly cost of 2380 RON.

**Table 2:** Estimated costs over 10 years for scenario A

**Group of patient**	**A1**	**A2**	**Max lost income**
HBP	119983.51	208467.46	41832.04
HBL	119983.51	208467.46	41832.04
HBS	97343.31	185827.26	41832.04
HBP and HBL	153943.81	242427.76	41832.04
HBP and HBS	131303.61	219787.56	41832.04
HBP and HBL and HBS	165263.92	253747.87	41832.04
**Average costs**	**131303.61**	**219787.56**	**41832.04**

Estimated costs over 10 years when the patient is not concerned about his/her heart health.

In the case of a permanent disability occurring as a consequence of a severe CVD, the above costs are supplemented as follows: 

– the loss of income from 790.1133 RON monthly (average income in Romania) to 512.96 RON monthly (the quantum of the disability pension in this case); and

– the attendant’s allowance, which is 512.96 RON monthly.

Thus, on average and taking into account the 5% CPI, the cost of a severe CVD is 131303.61 RON over 10 years in case A1 and 219787.56 RON over 10 years in case A2. The maximum lost income is 41832.04 RON over 10 years in case A2.

The average costs for case B, which are presented in Table 3, include the following: 

– 4 regular visits to the general practitioner over the first 1.5 years, estimated at 200 RON;

– routine analyses performed at the same times as the visits to the general practitioner, estimated at 160 RON; and

– cardioprotective medication, estimated at approximately 216.67 RON monthly for the first 1.5 years.

**Table 3:** Estimated costs over 10 years for scenario B

**Group of patient**	**B1**	**B2**	**B3**	**Max lost income**
HBP	25658.90	107336.95	182548.30	35557.24
HBL	25658.90	107336.95	182548.30	35557.24
HBS	10565.43	85828.75	161040.10	35557.24
HBP and HBL	48299.10	139599.23	214810.58	35557.24
HBP and HBS	33205.63	118091.04	193302.39	35557.24
HBP and HBL and HBS	55845.83	150353.33	225564.68	35557.24
**Average costs**	**33205.63**	**118091.04**	**193302.39**	**35557.24**

Estimated costs over 10 years when the patient is concerned about his/her heart health and performs proper prevention activities.

Thus, taking into account the CPI, the prevention costs are estimated to be approximately 4008.71 RON over the first 1.5 years. For the rest of the period (8.5 years), the average costs are as follows: 

– in case B1, when no severe CVD occurs, 29196.92 RON;

– in case B2, when one severe CVD with minor consequences occurs, 114082.33 RON; and

– in case B3, when a severe CVD with major consequences occurs, 189293.68 RON.

In the best case (B1), the 10-year prevention costs are 33205.63 RON (i.e., 25.29% of the minimal costs and 15.11% of the maximal costs generated by a severe CVD in the same period of time).

In the case when one severe CVD with minor consequences occurs (B2), the costs are 118091.04 RON over 10 years (i.e., 89.93% of the costs generated by a similar severe CVD in the same period of time).

In the case when one severe CVD with major consequences occurs (B3), the costs are 193302.39 RON over 10 years (i.e., 87.95% of the costs generated by a similar severe CVD in the same period of time). In this case, the maximum lost income over 10 years is 85% of the lost income in case A2.

Hence, with proper prevention, the total CVD costs are reduced by a percentage varying from 10.07% to 84.89%.

Table 4 presents the costs of treatment in the three cases, displayed in the following categories: total costs, patient-supported costs and state-supported costs. It can be seen that on average, the state-supported costs are 23.43% in case A1, 54.26% in case A2, 57.20% in case B1, 25.83% in case B2 and 54.69% in case B3. With proper prevention, the patient’s expenses decrease by an amount that ranges from 12.88% to 85.86% of the expenses incurred when no prevention activities were conducted. The state reduces its expenses at best by 87.07% (case B1 versus case A1) and between 0.86% and 11.35% for similar CVDs (i.e., B2 versus A1 and B3 versus A2).

**Table 4:** Patient-supported costs and state-supported costs over 10 years

**Scenario**	**Total costs**	**Patient-supported**	**State-supported**	**Patient’s income**	**Percent allocated for health**
A1	131303.61	100535.07	30768.54	119255.50	84.30%
A2	219787.56	100535.07	119252.49	77423.45	129.85%
**A (average)**	**175545.59**	**100535.07**	**75010.52**	**98339.48**	**102.23%**
B1	33205.63	14213.02	18992.61	119255.50	11.92%
B2	118091.04	87586.77	30504.27	119255.50	73.44%
B3	193302.39	87586.77	105715.62	75106.09	116.62%
**B (average)**	**114866.35**	**63128.85**	**52070.83**	**104539.03**	**60.39%**
**Average costs**	**139138.05**	**78091.34**	**61246.71**	**102059.21**	**76.52%**

Patient-supported costs, state-supported costs and the ratio of the patient’s income allocated for heart health for the scenarios considered above.

#### Income ratio allocated for health

The most recent available data [19] report that the average monthly earning in Romania is 790.1133 RON and the monthly pension for a total disability is 512.96 RON. Table 4 also contains the estimated 10-year income for the considered scenarios and the ratio of income allocated for health.

For the A scenarios (the unconcerned patient), the ratio of income allocated to health varies from 84.30% in case A1 to 129.85% in case A2. The latter case also accounted for the loss of income due to permanent disability. Thus, case A2 implies that the amount spent by the patient on his/her health exceeds his/her income, which leads to poverty. On average, the unconcerned patient spends 102.23% of his/her income on healthcare - again, more than his/her income. Thus, the average unconcerned patient faces poverty in the case of a severe CVD.

For the B scenarios (the concerned patient who is interested in preventing CVD), the ratio of income allocated for health varies from 11.92% in case B1 to 73.44% in case B2 and 116.62% in case B3. It can be seen that even if the patient is concerned about his/her heart health, the worst case scenario can also lead to poverty. However, an average concerned patient spends only 76.52% of his/her income on healthcare and thus it is more likely that he/she does not face poverty as a result of healthcare expenditures.

As a general trend, it can be observed that the ratio of personal income allocated to heart health decreases as the patient’s level of concern about heart health increases. Hence, the probability of facing poverty decreases as the patient’s level of concern about heart health increases.

### Reducing the Heart SCORE and costs through primary care

A total of 235 patients improved or maintained their SCORE factor by following either the rehabilitation program or the medication regimen recommended. These patients represent 91.43% of the patients for whom the SCORE factor did not rise. When improved, the SCORE factor improved on average by 48.05%.

**Figure 2:**
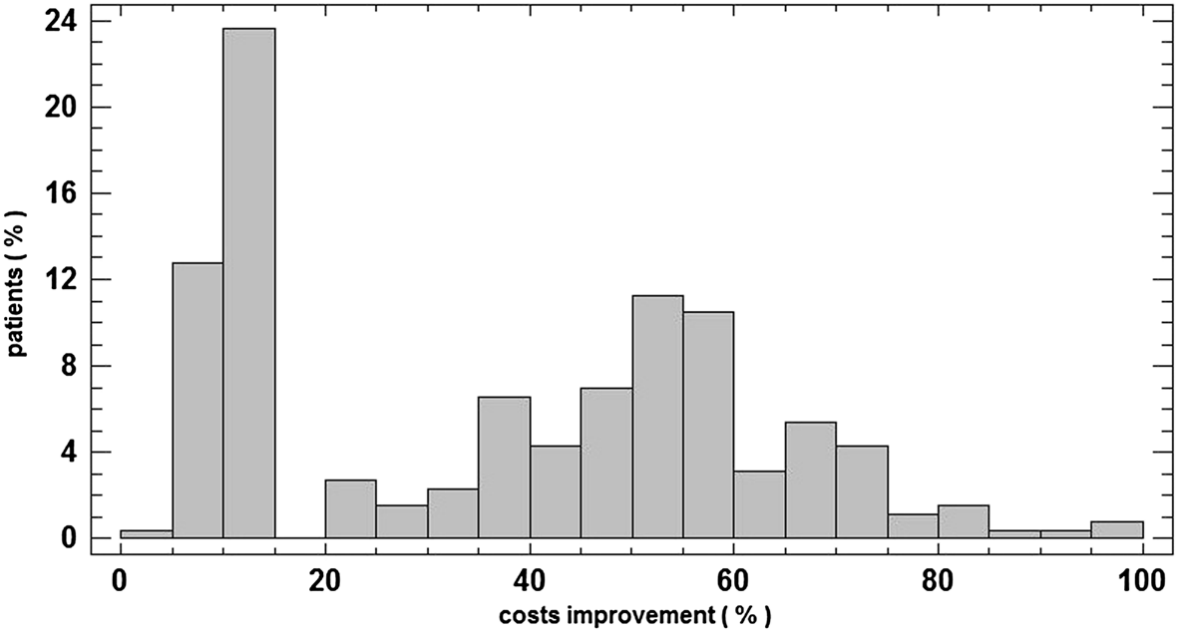
**Cost improvement.** Cost improvement (in percentages) for the patients who followed the recommendations.

The standard costs were reduced in 258 patients, 235 of whom either followed the rehabilitation program or complied with the recommended medication regimen, representing 91.08% of the patients who experienced reduced costs. The estimated costs improved on average by 37.26% (see Figure 2).

**Figure 3:**
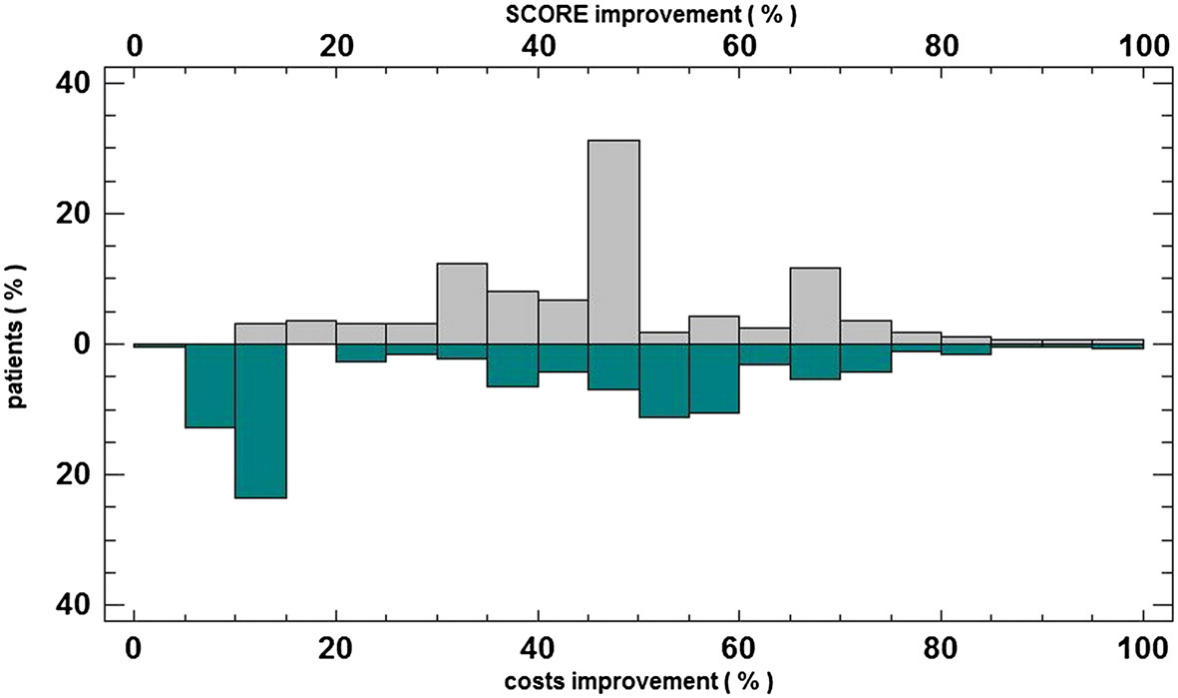
**SCORE improvement vs. cost improvement.** SCORE improvement versus cost improvement for patients who followed the recommendations.

Figure 3 shows a comparison between the improvement in costs and the improvement in the SCORE factor. While most of the patients improved their SCORE factor between 45% and 50%, the costs were improved at most by 10% to 15%.

### Reducing the risk of CVDs by primary care

These findings suggest that through proper primary care, even if the overall costs decrease significantly, the patient benefits more than the state. The previous sections of our analysis primarily highlighted the gains of the individual rather than the gains of state. Moreover, it seems that in the short term, the state encounters the same expenditures either by promoting prevention and primary care or by not promoting primary care and dealing only with the severe cases. However, is this really an advantage in the medium term and the long term?

To answer this question, we estimated two models for dealing with CVDs. These models (or scenarios) are based on the most recent data available and compute the number of patients that can be treated yearly, assuming that the healthcare budget is maintained at the same level as that of 2010. The goal of each model is the minimisation of the CVD-related morbidity rate to less than 4000 cases per 100000 population.

**Figure 4:**
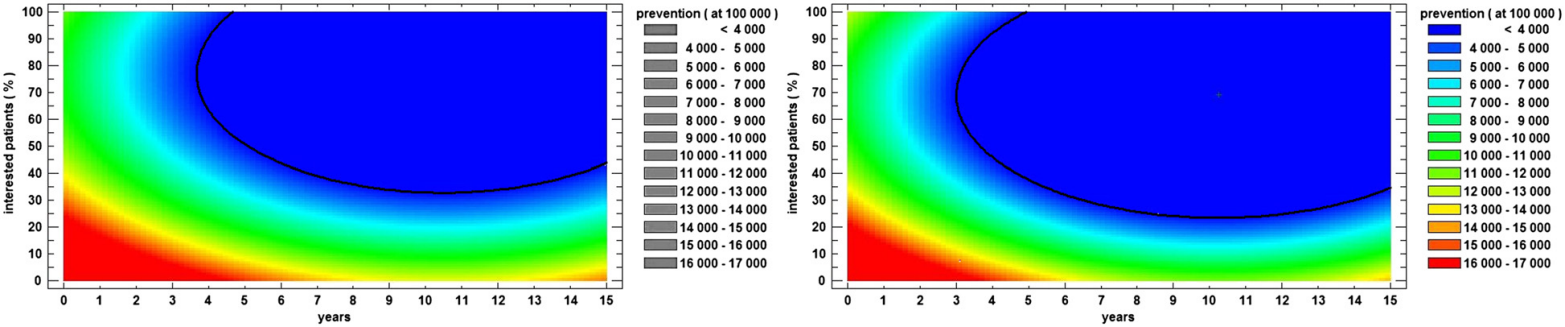
**Morbidity rate in two cases.** On the left, the morbidity rate in the “bad coverage scenario” and on the right, the morbidity rate in the “good coverage scenario” are presented.

Figure 4 shows the evolution of the morbidity rate for the two coverage scenarios considered. The goal is to reach the regions with less than 4000 cases of disease per 100000 population (depicted in blue). In the bad coverage scenario, for 30% to 40% of the population, proper prevention techniques lead to the goal in approximately 7 years. For between 50% and 60% of the population, the goal can be achieved in approximately 5 years. In the good coverage scenario, using the same percentages as previously considered yields a duration until the goal is achieved of approximately 5 years for 30%- 40% of the population and 4 years for 50%-60% of the population.

**Figure 5:**
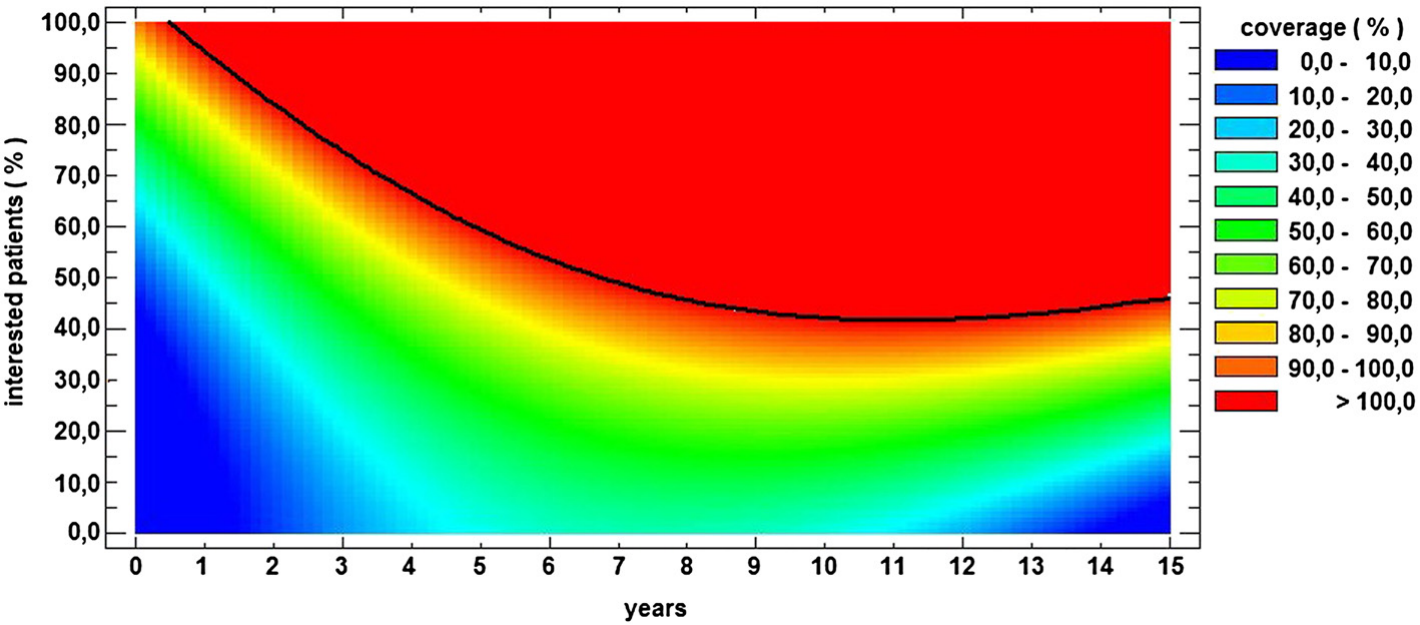
**Number of patients treated when no prevention was provided.** The largest number of patients is considered at the 0 moment of time and when 0% of the patients are engaged in prevention-that is, the number of patients with CVDs existing in 2010 in Romania.

**Figure 6:**
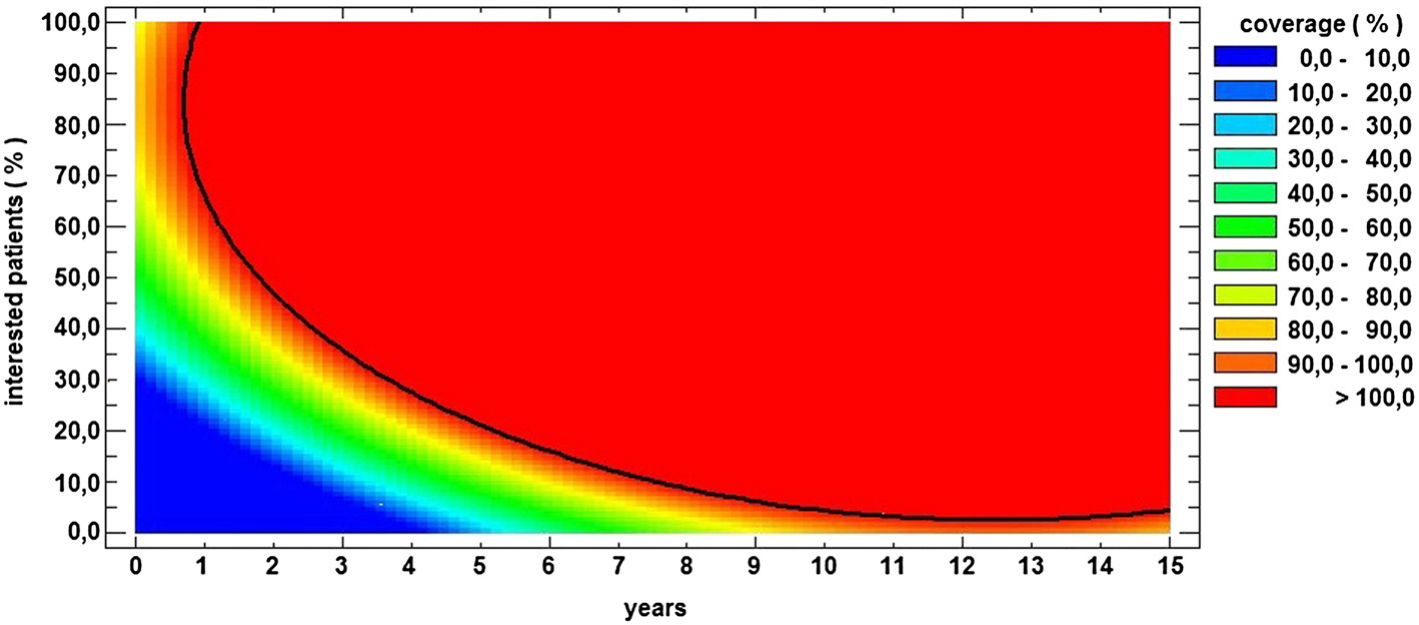
**Number of patients treated when prevention was provided.** The largest number of patients is considered at the 0 moment of time and when 0% of the patients are engaged in prevention-that is, the number of patients with CVDs existing in 2010 in Romania.

Figures 5 and 6 estimate the proportion of patients that could be treated from the healthcare budget allocated to CVDs in case of a severe CVD, assuming that the state’s expenses for a CVD patient are the averages depicted in Table 4. This coverage rate depends not only on the percentage of the patients interested in performing prevention at the current moment but also on the amount of time (starting from the current moment) during which the patients follow a prevention program. The goal is to reach 100% coverage, meaning that all the patients can be treated from the allocated healthcare budget (i.e., the red region). Values over 100% indicate that the number of existing patients is less than the number of patients with severe CVD that can be treated. Values under 100% indicate that not all of the existing patients with severe CVD can be treated without subjecting the health budget to supplementary expenditures (i.e., credits). Using a statistical software [30], the following mathematical expressions for the coverage rate were deduced:

- for the “bad coverage scenario”

$$\begin{array}{l} \;C(\%)=-17.363+12.9086\cdot Y\;-\;0.164764\cdot P-0.842294\cdot Y^{2}\\ \;+0.137817\cdot Y\cdot P+0.012122\cdot P^{2} \end{array}$$

- for the “good coverage scenario”

$$\begin{array}{l} \;C(\%)=-114.747+32.5884\cdot Y+4.56648\cdot P-1.30542\cdot Y^{2}\\ \;-0.0270339\cdot P^{2} \end{array}$$

where *C* represents the coverage rate, *Y* the duration in years (which is between 0 and 15 in our case) and *P* the percentage of the population currently interested (“year zero”) in performing proper primary care.

By solving the equation *C*=100, in the case of the “bad coverage scenario” (Figure 5), the following can be deduced: 

•if the percentage of the population that is interested in following proper primary care recommendations is less than 41.63%, then the healthcare budget will not cover all the severe cases during the next 15 years; and

•if the percentage of the population that is interested in following proper primary care recommendations is 41.63%, then the maximum duration of time until the proposed goal is achieved is 11.07 years.

By solving the equation *C*=100, in the case of the “good coverage scenario” (Figure 6), the following can be deduced: 

•if the percentage of the population that is interested in following proper primary care recommendations is less than 2.52%, then the healthcare budget will not cover all the severe cases during the next 15 years;

•if the percentage of the population that is interested in following proper primary care recommendations is 2.52%, then the maximum duration of time until the proposed goal is achieved is 12.48 years; and

•if the percentage of the population that is interested in following proper primary care recommendations is 41.63%, then in the “good coverage scenario”, it will take only 2.42 years to achieve the proposed goal (i.e., 4.56 times less than in the “bad coverage scenario”).

**Figure 7:**
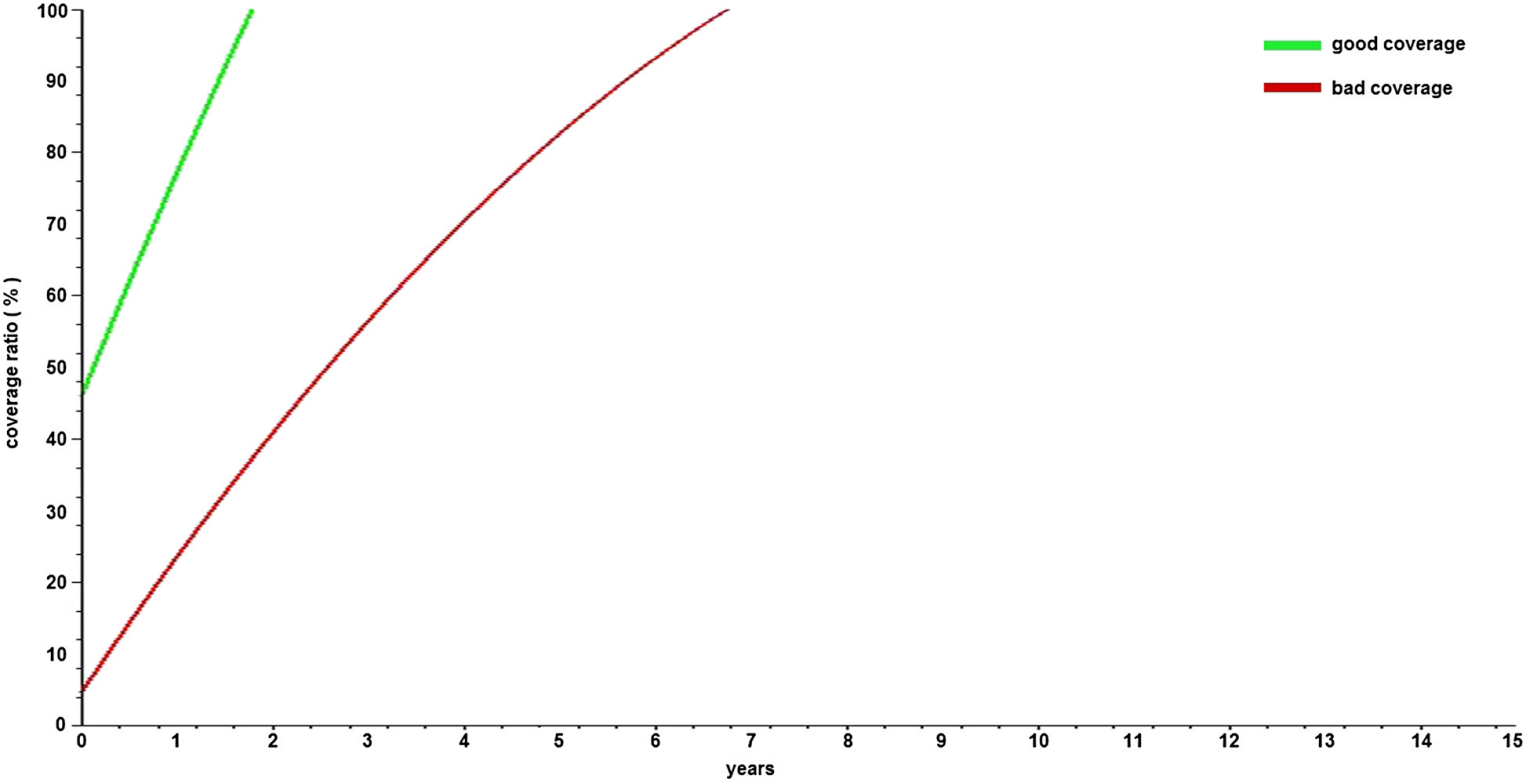
**CVD coverage from the healthcare budget for a 50% rate of prevention.** The CVD coverage by the healthcare budget is presented assuming 50% of the patients are performing proper prevention activities at the initial moment of time.

**Figure 8:**
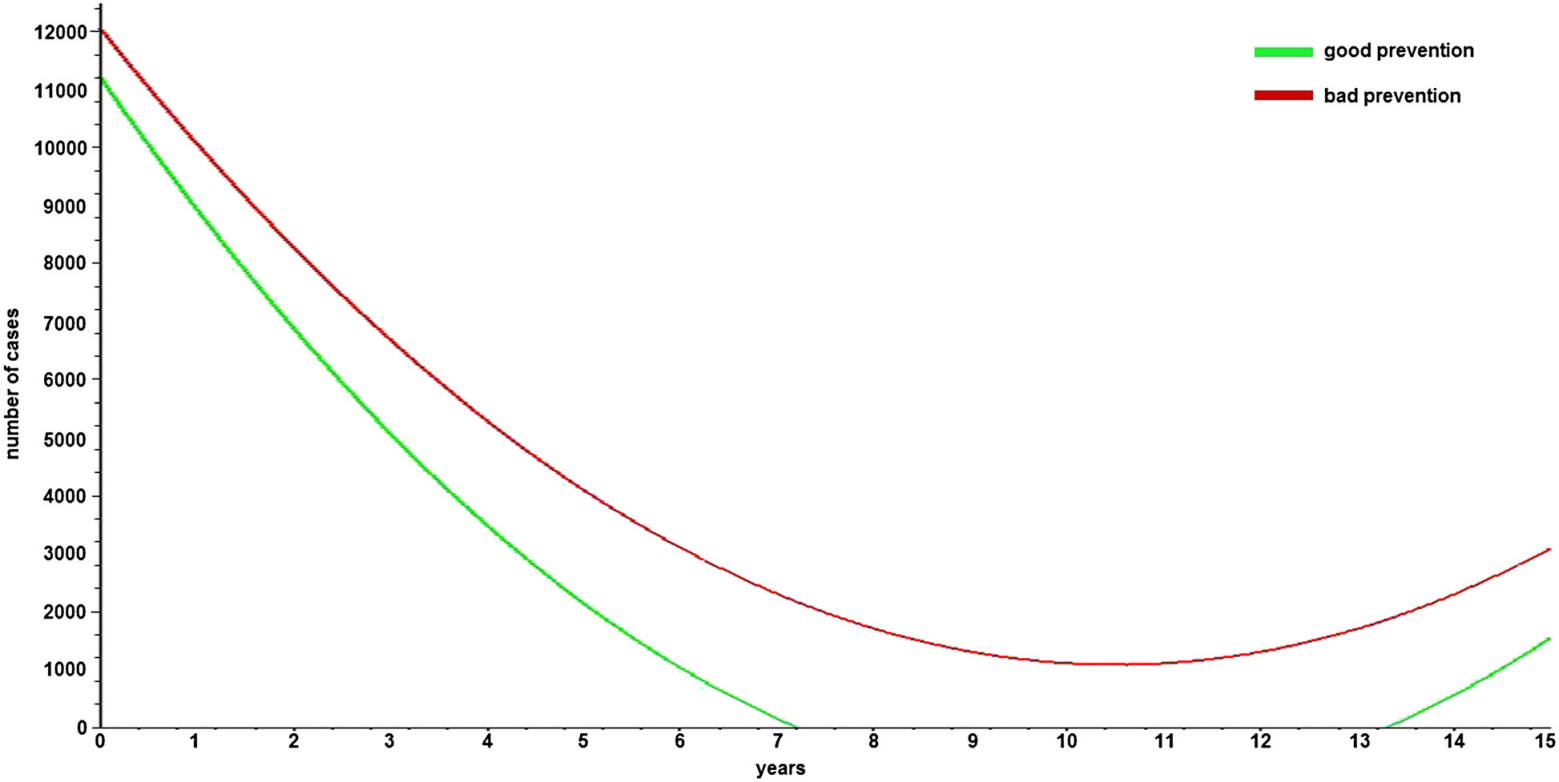
**Comparison between scenarios for a 50% rate of prevention.** The evolution of CVD-related morbidity is presented assuming 50% of the patients are performing proper prevention activities at the initial moment of time.

If the percentage of the population that is interested in following proper primary care recommendations is 50%, then the proposed goal will be achieved in 6.75 years in the “bad coverage scenario” and in only 1.78 years in the “good coverage scenario” (i.e., 3.78 times faster) (Figure 7). A similar comparison is performed with the estimated values for the morbidity rate, which in the case of the “good coverage scenario” leads to the proposed goal (less than 4000 cases per 100000 population) almost twice as fast as in the “bad coverage scenario” (Figure 8).

## Conclusions

Based on the available data, we estimated the costs of CVDs both for the state and for the patient. These estimations were performed for “best-case” and “worst-case” scenarios, depending on the patient’s degree of interest regarding his/her heart health. Along the limitations imposed by the lack of available medical data for Romanian patients, there are some other limitations of the present work that could be object to future research. A limitation of this study is that in both cases, it estimates the lowest costs involved; moreover, the health budget is assumed to be constant over a period of 10 years (which is a rough approximation, since the health budget depends on economic changes). It was assumed that all the patients benefit from health insurance (which is not the real case in Romania) and that their income does not change after suffering a CVD with minor consequences. It was also assumed that the patients follow all the recommendations of their general practitioner or specialist after suffering a severe CVD (which is not always the case) and because the state supports a percentage of the costs of the least expensive medication, it was assumed that the patients take the least expensive medication (which is also not always the case). Moreover, it was assumed that the patients do not suffer from any other form of disease during the 10-year period, such as flu, fractures, cancer or other forms of CVD (e.g., peripheral vascular diseases). Although lost productivity is a real issue highlighted by physicians, it could not be properly estimated due to the lack of economic data. These estimations are affected by the lack of available data concerning the economic burden of CVD in Romania, especially for the income lost due to illness and the real expenses for cardioprotective medications.

We found that when performed well, primary care not only reduces the risk of CVD but also reduces the costs of heart healthcare. A patient who is concerned about his/her heart health spends less of his/her income on heart health than an unconcerned patient. Moreover, the likelihood of facing poverty due to a CVD is less for a concerned patient than for an unconcerned patient.

An important result is that proper primary care has the long-term effect of reducing supplementary state expenditures for CVDs. While the short-term cost advantages for the state might not be spectacular, it is important to note that in the context of proper long-term primary care, the healthcare budget provides better coverage for severe CVDs and thus the supplementary expenditures are reduced.

Since the models for the expenses and coverage ratio for CVD patients were validated in Romania (considered a representative Eastern European country), they could be applied to any other Eastern European country, provided that the following data is available: health budget, state contribution to health expenses, prevention and treatment costs, average income of the population.

During our research, we observed a lack of interdisciplinary studies (i.e., medical and economic) concerning Romania (either as a stand-alone country or as representative of Eastern European countries); therefore, further work will study the economic impact of additional medical problems on the average patient.

## Endnotes

^a^Along heart attack and stroke, CVDs also include peripheral vascular diseases, congenital heart diseases, cardiomyopathies, arrhythmias [1]. However, while there is little data concerning heart attack and stroke in Eastern European countries, the situation is even worse for the other CVDs. For instance, for documenting peripheral vascular diseases, lower limb doppler ultrasound examination or angiography is needed, investigations that are usually not included in the medical history of Romanian patients. Hence our article only considers the cases of heart attack and stroke.^b^Western European countries are: Austria, Belgium, France, Germany, Liechtenstein, Luxembourg, Monaco, Netherlands, Switzerland [2].^c^Eastern European countries are: Belarus, Bulgaria, Czech Republic, Hungary, Poland, Republic of Moldova, Romania, Russian Federation, Slovakia, Ukraine [2].^d^Even if the food prices grew constantly in Europe since 2002 [31], the food prices in Eastern European countries remain below the average value of EU, and well below the similar values of Western Europe [32]. Hence, it is not far-fetched to state that, in Eastern Europe is rather inexpensive to consume fruits and vegetables daily.

## Abbreviations

CVD: Cardiovascular diseases; EU: European union; HBP: Patients following antihypertensive medication; HBL: Patients following lipid-lowering medication; HBS: Patients following diabetes therapies; RON: Romanian currency; EUR: EURO currency

## Competing interests

The authors declare that they have no competing interests.

## Authors’ contributions

TS coordinated the research collective and performed the statistical simulations. CA participated in the EUROASPIRE Follow Up and provided medical information. GVM and DD developed the cost-related scenarios. AB and FMM developed the coverage-related scenarios. MAU provided expertise in the public finance field. All authors read and approved the final manuscript.

## Authors’ information

TS is a professor at the Politehnica University of Timisoara. In addition to his initial degree and PhD in engineering, he has added a degree and a PhD in economy. CA is a physician with a PhD in medicine and is currently teaching in the Physical Education and Sports Faculty at the West University of Timisoara. GVM has experience in management and computer science. AB is an expert in management and human resources with a PhD in management. DD is an expert in finance and banking with a PhD in economics. FMM is an expert in accounting, applied computer science. MAU is an expert in public finance with a PhD in public finance.

## Pre-publication history

The pre-publication history for this paper can be accessed here:

http://www.biomedcentral.com/1472-6963/13/75/prepub
